# Retraction: Leadership empowering behaviour as a predictor of employees’ psychological well-being: Evidence from a cross-sectional study among secondary school teachers in Kohat Division, Pakistan

**DOI:** 10.1371/journal.pone.0322665

**Published:** 2025-04-10

**Authors:** The PLOS One Editors

The *PLOS One* Editors retract this article [1] due to concerns about compliance with the PLOS Human Subjects Research policy.

Ethics approval documentation provided in post-publication discussion was not sufficient to confirm compliance with this policy as a co-author of this article [1] was a signatory of the document and PLOS identified potential competing interests between one other signatory and one or more of the article’s authors. This issue raises concerns about the independence of the committee responsible for ethical oversight of this study. The editors regret that this issue was not identified prior to publication.

QS did not agree with the retraction. MAS, SS, IH, AZK, RUK, MA, and IK either did not respond directly or could not be reached.
